# Spectral optical coherence tomography findings in patients with ocular toxoplasmosis: A case series study

**DOI:** 10.1016/j.amsu.2020.04.008

**Published:** 2020-04-24

**Authors:** Feriel Ammar, Ahmed Mahjoub, Nadia Ben Abdesslam, Leila Knani, Mohamed Ghorbel, Hachmi Mahjoub

**Affiliations:** Department of Ophthalmology, Farhat Hached Hospital, Sousse, Tunisia

**Keywords:** Spectral optical coherence tomography, Ocular toxoplasmosis, Uveitis, SS-OCT, Swept-source optical coherence tomography, SD-OCT, Spectral domain optical coherence tomography, RPE, Retinal pigment epithelium, EZ, Ellipsoid zone

## Abstract

**Purpose:**

Ocular toxoplasmosis is the most common cause of infectious uveitis worldwide. The diagnosis of ocular toxoplasmosis is primarily clinical when it is a typical presentation.With an atypical presentation in the fundus, parasitological diagnosis is a decisive contribution, as well as multimodal imaging. The aim of this study was to investigate vitreal, retinal, and choroidal morphologic changes in active and scarred toxoplasmosis lesions using swept source optical coherence tomography. To our knowledge, it is the first study in Tunisia which describes with precision the retinochoroidal lesions caused by *Toxoplasma Gondi* by means of the optical coherence tomography (OCT).

**Methods:**

A retrospective analysis of fifteen patients diagnosed with ocular toxoplasmosis was conducted. The patients were examined at ophthalmology service of Farhat Hached Hospital in Sousse Tunisia between January 2002 and December 2019. Complete ophthalmologic examination including best-corrected visual acuity, slit lamp biomicroscopy, dilated biomicroscopic and fundus examinations, colour fundus photography as well as fluorescein angiography and OCT were done at the initial visit and during follow-up.

**Result:**

In the acute phase, thickening, hyper-reflectivity of the neurosensory retina, posterior shading, bumping of the RPE, hyporeflectivity and thickening of choroid were found in 86,6% of patients. During follow-up, neurosensory retinal layers thinning and disorganization, interrupting ofthe ellipsoid zone (EZ), and RPE hyper reflective were noticed in 73% of patients. The choroid became thin and more hyperreflective in 73% of patients. Multiple hyperreflective dots in the vitreous cavity and posterior hyaloid thickening were demonstrated in the acute phase in 60% of patients, with complete resolution and detachment of the posterior hyaloid in the scarred lesions.

**Conclusion:**

The SS-OCT is an important adjunctive imaging modality in the diagnosis and follow-up of patients with ocular toxoplasmosis.

## Highlights

• Ocular toxoplasmosis is the most common cause of infectious uveitis worldwide.
• The introduction of SD-OCT has enabled imaging with greater resolution, wider sampling area, and improved image registration.
• The active retinochoroiditis lesion shows thickening with hyper reflectivity of the internal layers of the retina.
• The retinochoroidal scar shows retinal atrophy, interruption of the IS/OS line, sometimes subretinal fibrosis.

## 1 Introduction

Ocular toxoplasmosis is the most common cause of infectious uveitis worldwide with a prevalence ranging from 3.8% to 17.7% [1]. It is the leading cause of posterior uveitis (38.3%) in Tunisia [2]. Indeed, the eye is the main target organ for symptomatic manifestations of the infection, which may be congenital or acquired [3]. It most often affects young people between the ages of 20 and 40 [4]. The diagnosis of ocular toxoplasmosis is primarily clinical when it is a typical presentation [5]. It is presumed when discovering an evocative lesion in the eye fundus. Active, whitish, oedematous retinochoroiditis lesions are distinguished from cicatricial, pigmented and atrophic lesions. Active lesions result in contiguity hyalitis and sometimes anterior uveitis, of variable intensity (4). However, in many cases, chorioretinal lesions observed at the fundus are not typical and may be confused with lesions of other microorganisms [5]. With an atypical presentation of ocular toxoplasmosis in the fundus, parasitological diagnosis is a decisive contribution, as well as multimodal imaging.

Swept-source optical coherence tomography (SS-OCT) is the latest milestone in retinal and choroidal imaging. Because its wavelength of 1050 nm, which is superior to the 840 nm of spectral domain optical coherence tomography (SD-OCT), it is able to overcome ocular opacities such as cataracts and vitritis, wich allows retinal and choroidal visualization of eyes whose fundus is not clearly visible. Consequently, SS-OCT allows visualization of the retinal and choroidal vascular networks, even in eyes with medium opacity [6].

The aim of our study was to investigate vitreal, retinal, and choroidal morphologic changes in active and scarred toxoplasmosis lesions using SS-OCT. To our knowledge, it is the first study in Tunisia which describes with precision the retinochoroidal lesions caused by *Toxoplasma Gondi* by means of the optical coherence tomography (OCT).This manuscript is reported in ac-cordance with PROCESS guidelines [15].

## 2 Methods

Fifteen eyes of fifteen patients consecutively diagnosed with active ocular toxoplasmosis were included in this retrospective study. The patients were examined by the medical staff (i.e. residents and seniors) at the ophthalmology service of university hospital center of Farhat Hached in Sousse Tunisia between January 2002 and December 2019. One patient had amblyopia, two patients had hypertension, and two other patients had diabetes. The diagnosis was based on the criteria described by Holland and colleagues (3): the presence of an active white focal retinal lesion, with or without associated hyperpigmented chorioretinal scars, and confirmed by laboratory studies. . Complete ophthalmologic examination including best-corrected visual acuity, slit lamp biomicroscopy, dilated biomicroscopic and fundus examinations, colour fundus photography as well as fluorescein angiography and OCT were done at the initial visit and during follow-up. Retina and choroid OCT protocols directed to macular area and lesions observed on clinical examination were used.

## 3 Results

**Fig. 1:**
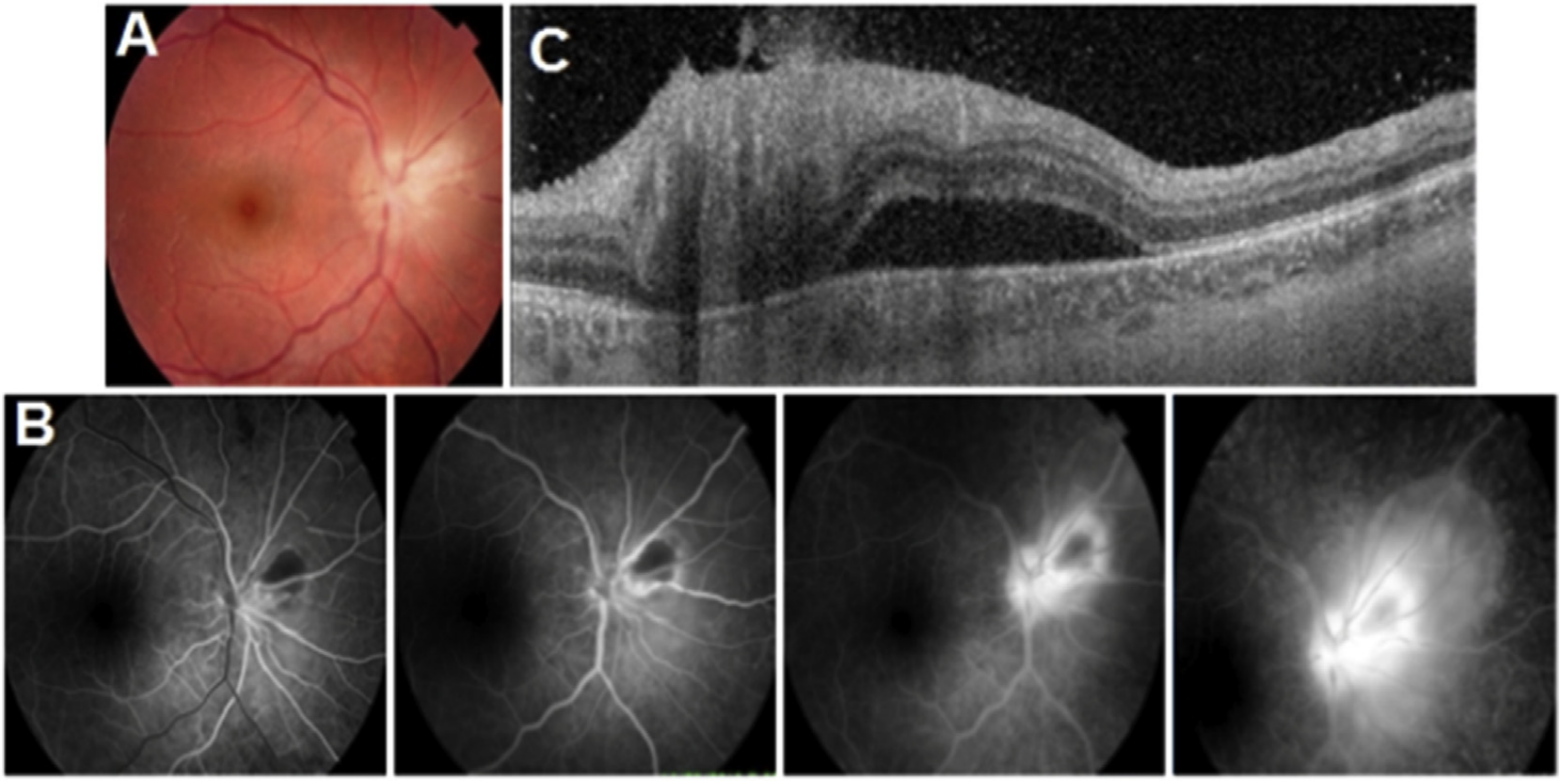
(A) Fundus Photo: Juxta papillary active retinochoroiditis (Jensen). (B) Angiographic sequence of Jensen's retinochoroiditis: centripetal hyperfluorescence of the edges of the active lesion. To note that the serous retinal detachment is best seen in the late phase. (C) OCT: hyper-reflectivity and thickening of the inner layers of the retina, hyper-reflective vitreous points, and serous retinal detachment.

**Fig. 2:**
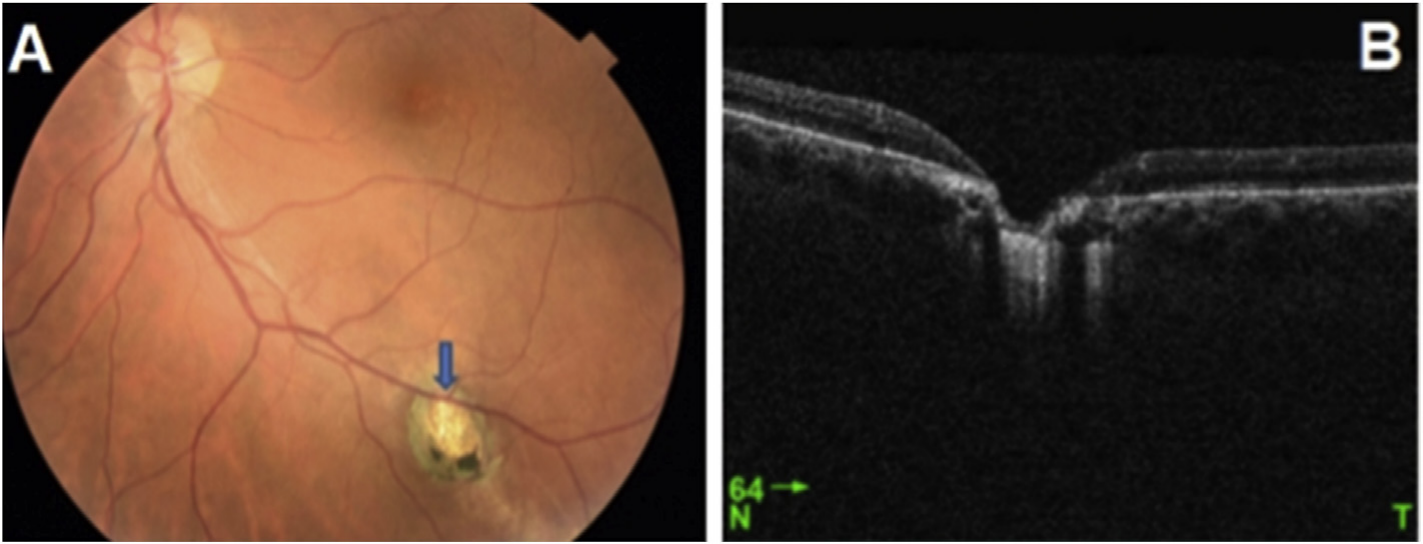
(A) Scarred lesion next to the lower temporal arch. (B) OCT of the cicatricial lesion showing a thinning of the neurosensory retina, an interruption of the EZ, a thinned and hyper reflective choroid.

Seven active lesions and five retinochoroidal scars were studied. In the acute phase (Fig. 1), thickening, hyper-reflectivity of the neurosensory retina, posterior shading and bumping of the retinal pigment epithelium (RPE) were found. The choroid became thickened and hyporeflective (Table 1). The choroidal hyporeflectivity may be partially related to the hyperreflectivity of the retinal deeper layers. During follow-up (Fig. 2), neurosensory retinal layers thinning and disorganization, interrupting of the ellipsoid zone (EZ), and RPE hyper reflective were noticed. The choroid became thin and more hyperreflective (Table 2). Multiple hyperreflective dots in the vitreous cavity, compatible with vitritis, and posterior hyaloid thickening were demonstrated in the acute phase, with complete resolution and detachment of the posterior hyaloid in the scarred lesions. The hyperreflective dots became smaller, and resolved during follow-up. The sclera beneath the scar was hyperereflective and demarcated the scar borders. Epiretinal membrane was found over one active lesion (see Table 3).

**Table 1:** Active retinochoroiditis (13 cases).

OCT of active lesion	Number of cases	Proportion/hole population
**Retinal thickening**	13/13	86,6%
**Hyper-reflectivity of the inner layers of the retina**	13/13	86,6%
**Loss of retinal striations**	13/13	86,6%
**Bumping of the RPE**	12/13	80%
**Interrupting of the EZ**	11/13	73,3%
**Posterior shading**	13/13	86,6%
**Hyper-reflective dots in the vitreous cavity**	9/13	60%
**Posterior hyaloid thickening**	9/13	60%
**Degree of attachment of the posterior hyaloid Attached**	4/13	26,6%
**Partly Attached**	6/13	40%
**Detached**	3/13	20%
**Choroidal thickening**	11/13	73,3%
**Choroidal hypo-reflectivity**	13/13	86,6%

**Table 2:** Retinochoroidal scars (11 cases).

OCT of scarred lesion	Number of cases	Proportion/hole population
**Thinning and disorganization of the neurosensory retina**	11/11	73,3%
**Thin and hyper-reflective choroid**	11/11	73,3%
**Hyper-reflective fibrosis ahead the RPE**	4/11	26,6%
**Interrupting of the EZ**	11/11	73,3%
**Hyper-reflective dots in the vitreous cavity**	_	
**Posterior hyaloid thickening**	4/11	26,6%
**Degree of attachment of the posterior hyaloid Attached**	_	
**Partly Attached**	6/11	40%
**Detached**	5/11	33,3%
**Choroidal hyper-reflectivity**	11/11	73,3%

**Table 3:** Macular OCT (15 cases).

Macular OCT	Number of cases	Proportion/hole population
**Macular edema Cystoid**	3/15	20%
**Non-cystoid**	5/15	33,3%
**Serous retinal detachment**	6/15	40%
**Epiretinal membrane**	3/15	20%
**Macular atrophy**	2/15	13,3%

## 4 Discussion

The diagnosis of ocular toxoplasmosis is essentially based on the presence of characteristic clinical findings, including focal retinochoroiditis, an adjacent or nearby retinochoroidal scar, and moderate to severe vitreous inflammation [7]. OCT is a noninvasive imaging modality that provides high-resolution, cross-sectional images of the retina. The recent introduction of SD-OCT has enabled imaging with greater resolution, higher scan speed, wider sampling area, and improved image registration [8].

Some of the SD-OCT findings in ocular toxoplasmosis have been described previously (9,10); it included an increased reflectivity in the inner retina; a shadowing of the outer retina layers; and a thickened and detached posterior hyaloid with irregular hyperreflective formations. During follow up, it has been noted a separation of the posterior hyaloid, epiretinal formation and increased reflectivity of the inner retinal layers. Cho and Nam [9] reported increased intraretinal reflectivity corresponding to the area of retinitis with shadowing of the underlying choroidal tissue. Posterior hyaloid thickening and detachment over the lesion and contained irregular hyperreflective formation have been also noted [11].

In this study, we examined the vitreal, retinal, and choroidal morphological changes of ocular toxoplasmosis, during the acute and resolved phases, using SS-OCT. Similar changes were noticed in all eyes at various disease phases. The active retinochoroiditis lesion shows thickening with hyper reflectivity and disorganization of the internal layers of the retina associated with shading of the retinal-choriocapillary pigment epithelium complex [[10], [11], [12]]. There is often an interruption of the EZ, a bumping and a discontinuity of the RPE. Choroidal thickening under the lesion with choroidal hypo reflectivity is often observed. OCT also shows a thickening of the posterior hyaloid that can be totally or partially detached, adherent or not to the lesion, with the presence of hyper-reflective dots the vitreoretinal interface and cells at the vitreous cavity [9,10,13]. OCT passing through the retinochoroidal scar usually shows retinal atrophy, interruption of the EZ, sometimes subretinal fibrosis and discontinuity of RPE more or less associated with posterior hyaloid detachment. Posterior reinforcement of the choroid is usually noted [10,13]. OCT identifies potential complications including epi-retinal membrane, cystoid macular edema, retinal serous detachment, vitreoretinal traction, and choroidal neovascularization [[10], [11], [12]].

OCT is a safe and non invasive method, therefore follow-up measurements are unlimited, while fluorescein angiography is invasive and it carries, in certain subjects, some risks and side effects. Active toxoplasmic lesions have shown 3 main OCT characteristics wich are a highly reflective intraretinal layers corresponding with the retinitis area, a posterior hyaloid thickening and detachment over the lesion, and a shadow effect of the choroidal tissue. Reliable measurements of retinal thickness by OCT may be impossible in the presence of severe vitritis. Fluorescein angiograms is still very useful for the assessment of vasculitis and blood-retinal barrier breakdowns, while OCT detects sub-retinal fluid with a higher sensitivity [14]. Our study expose that OCT imaging can distinguish between active lesions and scars in ocular toxoplasmosis. OCT affords quantitative measurements of retinal thickness, which could be helpful in future prospective studies, to guide therapeutic decisions and to supervise the efficacy of treatments.

## 5 Conclusion

The findings of this study corroborate the hypothesis that toxoplasma has a tropism for neural tissues; it begins in the retina, and the inflammation may involve the entire retina as well as the choroid and vitreous. OCT can show whether the infection is in the acute phase or becoming quiescent. The SS-OCT is an important adjunctive imaging modality in the diagnosis and follow-up of patients with ocular toxoplasmosis. It is recommended to carry out other prospective studies on SS OCT aspects of toxoplasmic retinochoroiditis in a larger sample of patients.

## CRediT authorship contribution statement

**Feriel Ammar:** Data curation, Writing - review & editing. **Ahmed Mahjoub:** Data curation, Writing - review & editing. **Nadia Ben Abdesslam:** Writing - review & editing, Supervision. **Leila Knani:** Data curation, Writing - review & editing. **Mohamed Ghorbel:** Supervision. **Hachmi Mahjoub:** Supervision.

## Declaration of competing interest

The authors have no conflict of interest to declare.

## Registration of research studies

Name of the registry: clinicaltrials.gov.

Unique Identifying number or registration ID: U23454.

Hyperlink to the registration (must be publicly accessible): https://clinicaltrials.gov/ct2/show/NCT04219176.
